# Chirality induced long-range spin-selective transport in helical 3D metal–organic frameworks

**DOI:** 10.1039/d6sc01358a

**Published:** 2026-04-14

**Authors:** Pravesh Singh Bisht, Rabia Garg, Tapan Kumar Das, Nidhi Bhatt, Subash Chandra Sahoo, Amit Kumar Mondal

**Affiliations:** a Institute of Nano Science and Technology (INST) Mohali, Sector 81 Sahibzada Ajit Singh Nagar Punjab 140306 India amit@inst.ac.in; b Department of Chemical and Biological Physics, Weizmann Institute of Science Rehovot 7610001 Israel; c Department of Chemistry, Panjab University Chandigarh Sector 14 Chandigarh 160014 India

## Abstract

The exploration of novel chiral molecules as efficient spin-filtering materials *via* the chirality induced spin selectivity (CISS) effect remains an active and evolving area of research within the field of chiral spintronics. Previous studies that evaluated efficient spin filtering materials primarily focused on the degree of spin polarization achieved, often overlooking critical factors such as current intensity and the range of electron conduction. In this work, we endeavoured to provide a balanced assessment of these three parameters, and consequently, we reported highly efficient spin filtering materials based on the CISS effect in three-dimensional (3D) chiral metal–organic frameworks (MOFs). A significant aspect of this work is the successful demonstration of the CISS effect in two homochiral MOF crystals, where we achieved precise control over their high degree of spin-selectivity, a long range of spin filtering (>1 µm), and substantial current intensity (∼130 nA). This is attributed to their opposite helical structures and multidimensional electron transport channels. Notably, this study provides the first demonstration of spin-selective transport in transition metal-based helical 3D MOFs. To further validate the spin-dependent transport process in chiral 3D MOFs, we developed a prototype device featuring a spin-valve configuration. Notably, the results obtained from this device correlate well with the spin-dependent processes and confirm the presence of the CISS effect in the studied MOF materials. Furthermore, we have established a direct correlation between the contact potential difference (CPD) and the chirality of the 3D MOF crystals, offering new insights into their preferential spin transport properties *via* the CISS effect. This correlation, largely overlooked in previous studies, represents a key advance in understanding and designing efficient CISS-based spintronic materials.

## Abstract 2

In this study, we have demonstrated chirality induced long-range spin-selective transport in three-dimensional (3D) chiral metal–organic frameworks (MOFs) through the CISS effect.

## Graphical abstract

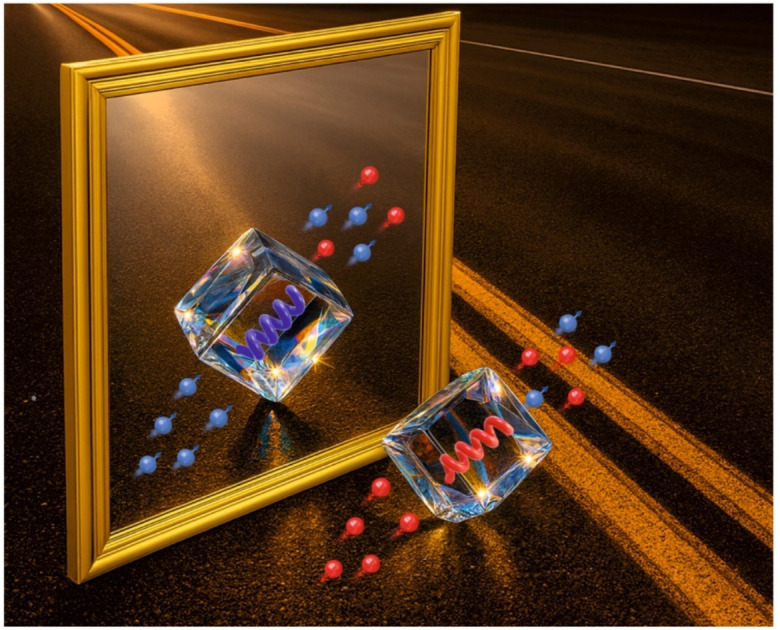

## Introduction

Techniques for generating spin current, as outlined in the literature, are predominantly confined to magnetically active materials and spin-active interfaces.^1^ Among these, transition metal ferromagnets and their alloys demonstrate moderate spin polarization (SP) capabilities, thus limiting the spin-dependent effects such as magnetoresistance, spin accumulation, *etc.*^2,3^ Recently, it has been possible to selectively extract and utilize spin-polarized electrons through the use of chiral compounds. This progress stems from the discovery of the chiral-induced spin selectivity (CISS) effect.^4–7^ Electrons possess an intrinsic quantum mechanical property called spin, which represents their intrinsic angular momentum. It can be viewed as a spinning top having two directions, either clockwise or anticlockwise often referred to as spin up or spin down.^8^ Chirality is the property of asymmetry of a molecule, resulting in the existence of non-superimposable mirror images of a molecule known as the enantiomers, typically designated as l-form (left-handed) and d-form (right-handed).^9^ When electrons propagate through the helical electrostatic potential of chiral molecules, the spinning motion of electrons gets coupled with its angular momentum *via* spin–orbit interactions along with the tunnelling mediated charge transport (fundamentally modified by decoherence), thus resulting in the preferential transmission of one spin over the other, determined by the handedness of the chiral molecule. This phenomenon is therefore referred to as the CISS effect and it has gathered renewed interest due to its remarkable ability to control the spin selective electron transmission using chiral materials, that too without the need of an external magnetic field.^10–17^ In light of the rapid developments in this field, a variety of chiral materials such as organic and hybrid systems,^18,19^ organic polymers,^20–23^ MOFs,^24,25^ overcrowded alkenes,^26,27^ and organic–inorganic perovskites,^28,29^ have been engineered for CISS. These materials serve as effective spin filters, successfully addressing challenges such as weak spin–orbit coupling (SOC) and exchange interactions.^30–32^

Despite significant advancements, the design of chiral materials that enable highly efficient spin polarized electron transport, along with long-range spin transport, remains a key challenge. In this study, we demonstrated that chiral metal–organic frameworks (MOFs) can function as an effective platform for harnessing the CISS effect to enable long range spin-selective electron transport. MOFs are crystalline materials formed from metal ions or clusters linked by organic linkers, that can be one-, two-, or three-dimensional.^33,34^ An important and evolving class of MOFs *i.e.* chiral MOFs are porous materials having asymmetric structures and are widely used in applications such as chiral transcription, enantiomeric separation, chiral catalysis, and more.^35,36^ The utilization of chiral MOFs for spin selective transport of electrons *via* the CISS effect is an emerging and underexplored area, which is still in its infancy stages. Building on our previous work, which successfully demonstrated spin selectivity in a purely achiral system through the spontaneous resolution technique to obtain random chiral MOF crystals,^24^ our current objective is to achieve precise control over spin selectivity as well as long-range spin transport by introducing homochirality in MOFs using chiral building blocks.

**Scheme 1:**
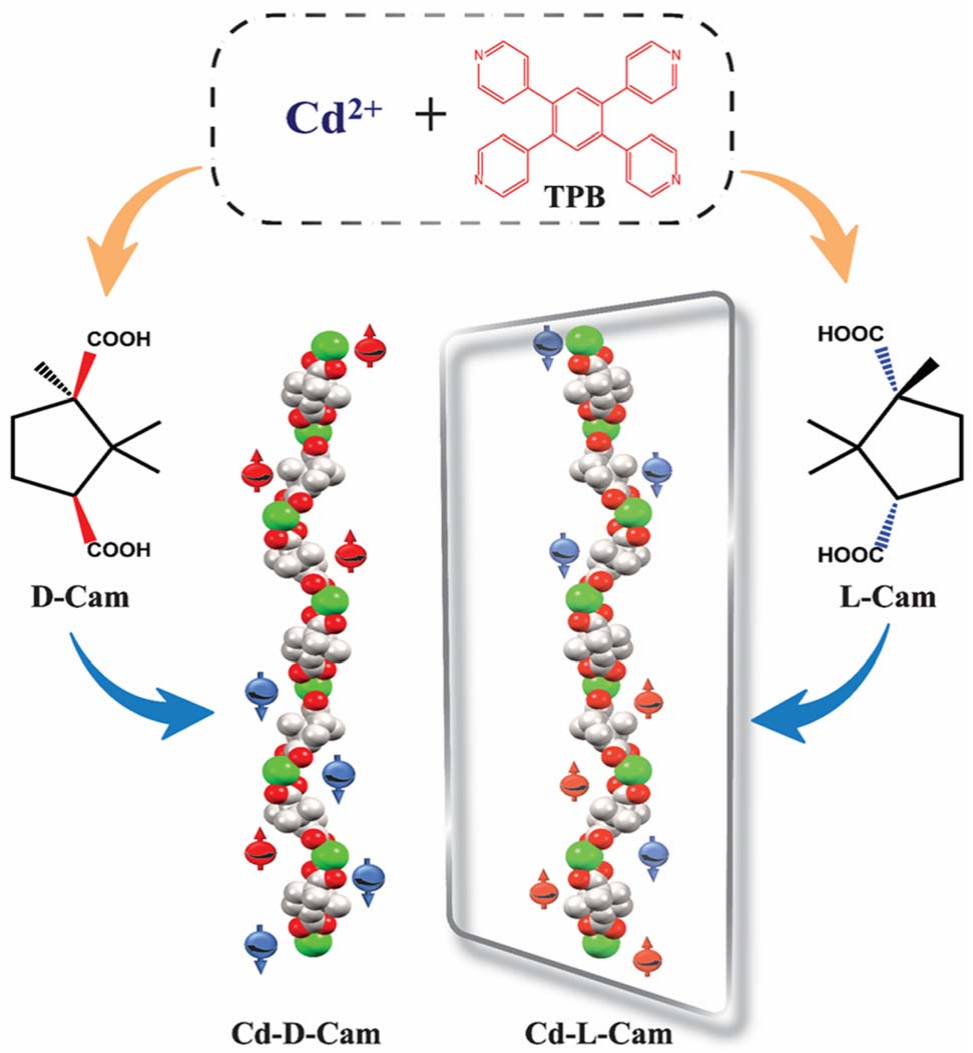
Schematic representation of the studied system and corresponding chirality induced spin dependent electron transfer processes.

Recently, an efficient 3D MOF spin-filter based on paramagnetic Dy^3+^ has been reported by Sebastian *et al.*^37^ However, the exploration of transition metal-based 3D MOFs within the CISS framework remains largely uncharted. To address this, our current work focuses on the utilization of 3D crystalline MOFs as a spin-filtering material, achieved through the self-assembly of the achiral molecule 1,2,4,5-tetra(pyridin-4-yl)benzene (TPB) and chiral camphoric acid (d/l-Cam) in the presence of Cd^2+^ ions (Scheme 1). The multidimensional propagation of chiral helices along different crystallographic directions within the MOF may promote multichannel electron transport. Additionally, the significant spin–orbit coupling (SOC) of the 4d transition metal Cd^2+^ centres can enhance the CISS effect. These combined factors likely contribute to the realization of long-range spin-polarized charge transport with high spin polarization (SP) values.

## Results and discussion

**Fig. 1:**
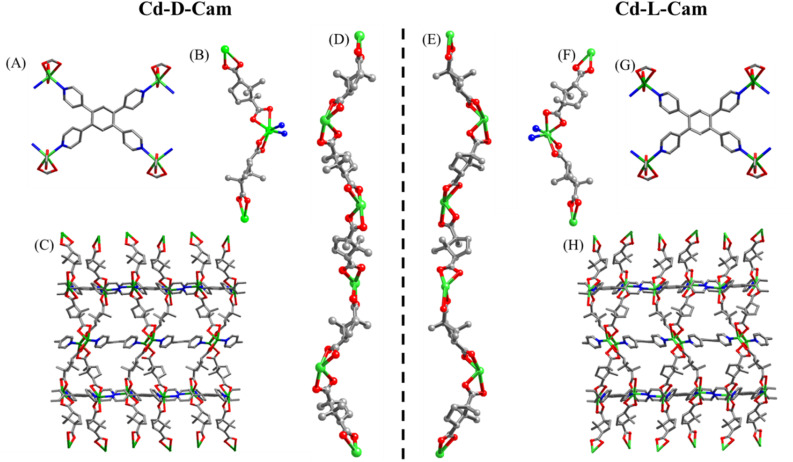
Structural descriptions of complexes, Cd-d-Cam and Cd-l-Cam. (A and G) The coordination mode of TPB ligands in Cd-d-Cam and Cd-l-Cam. (B and F) The coordination environment of Cd^2+^ ions in Cd-d-Cam and Cd-l-Cam. (C and H) The 3D sheet-like network along the bc-plane in Cd-d-Cam and Cd-l-Cam. (D and E) The 1D helical chains (P and M) consist of Cd^2+^ ions and d/l-Cam ligands along the *c*-axis. Color codes: Cd (green), O (red), N (blue), and C (gray).

Both the complexes (Cd-d-Cam and Cd-l-Cam) were synthesized *via* a solvothermal method involving Cd(NO_3_)_2_·4H_2_O, 1,2,4,5-tetra(pyridin-4-yl)benzene (TPB) and d/l-camphoric acid (d/l-Cam) in a solvent mixture of DMA and water according to a reported procedure (Fig. S1).^38^ These crystals have been characterized by single-crystal X-ray diffraction. The resulting compounds crystallize in a trigonal crystal system with space groups *P*3_1_21 and *P*3_2_21, respectively. Each Cd^2+^ ion forms a distorted tetrahedral structure by coordinating with two carboxylate groups from d/l-Cam and two pyridyl *N*-atoms of TPB (Fig. 1B and F). Considering only the interaction between Cd^2+^ ions and d/l-Cam ligands leads to the formation of a one-dimensional (1D) helical chain of each chiral type along the *c*-axis (Fig. 1D and E). The chirality of the camphoric acid ligand (d/l) plays a crucial role in directing the absolute helicity of the chain. The TPB ligand serves as a pivotal linker, connecting four Cd-(d/l)-Cam helical chains to construct a sheet-like network along the bc-plane, which is further vertically extended culminating in a three-dimensional (3D) homochiral framework for each Cd-compound (Fig. 1C and H). Although the overall structure is non-interpenetrated, it contains solvent-accessible voids occupied by lattice water molecules and diffused DMA molecules. These diffused DMA molecules could not be accurately assigned, hence masked. The crystallographic data and structural parameters were found to be consistent with the previous observations^38^ and are summarized in Tables S1 and S2. The corresponding bond lengths and bond angles around Cd^2+^ are given in Table S2.

**Fig. 2:**
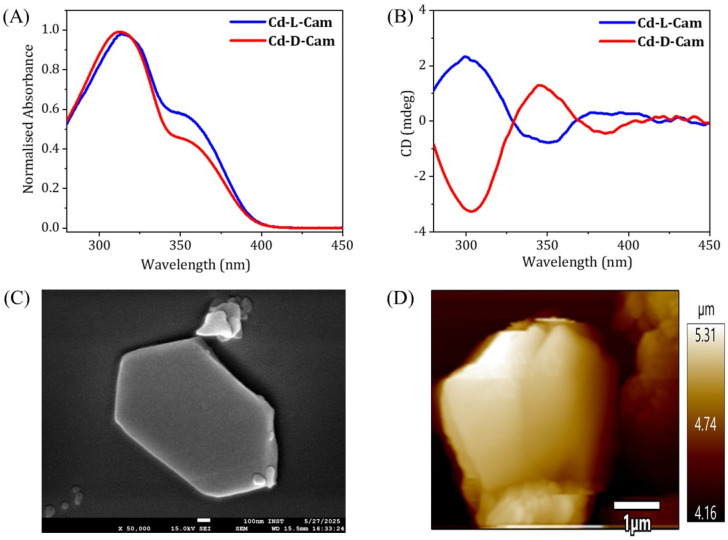
(A) Absorption spectra of Cd-d-Cam (red) and Cd-l-Cam (blue) crystals. (B) CD spectra of Cd-d-Cam (red) and Cd-l-Cam (blue) showing opposite signals. (C) High-resolution SEM image of a Cd-Cam single crystal. (D) AFM image of Cd-d-Cam crystal having a height of ∼900 nm as shown in the height profile image (Fig. S5).

To evaluate the chiroptical properties of these helical MOFs, UV-vis absorption and circular dichroism (CD) spectra have been recorded (Fig. 2A and B). Cd-d-Cam shows a positive Cotton effect at around 306 nm and Cd-l-Cam shows an opposite peak at around 305 nm, which confirms their chiral nature. The absorption band at around 305 nm has been observed due to the π–π* transition of TPB molecules. These results showed that chirality has been successfully induced in an overall architectural framework of the 3D MOF. The high-resolution images of these crystals have been obtained by using scanning electron microscopy (SEM) (Fig. 2C) and atomic force microscopy (AFM) (Fig. 2D). Their elemental analysis has been successfully confirmed by using the energy dispersive X-ray analysis (EDX) (Fig. S2) and X-ray photoelectron spectroscopy (XPS) (Fig. S3 and S4).

**Fig. 3:**
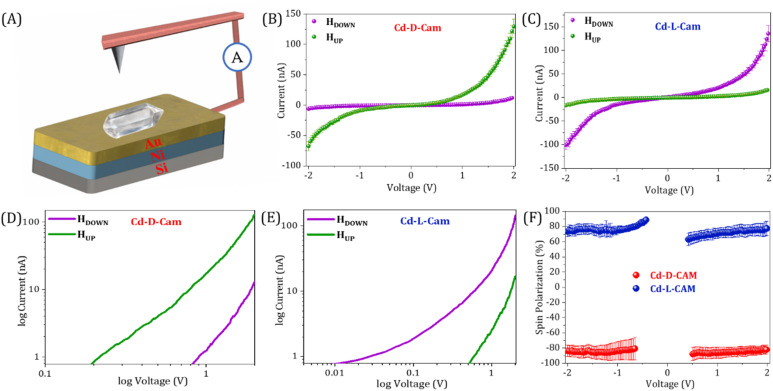
(A) Schematic representation of the mc-AFM setup used for the spin transport studies of 3D chiral MOFs on Ni/Au (100/8 nm). (B and C) The averaged current *versus* voltage (*I*–*V*) curves (with standard error) for Cd-d-Cam and Cd-l-Cam, respectively, with the Ni layer magnetized with the north pole pointing up (green) or down (purple). (D and E) Corresponding curves as a log–log plot for Cd-d-Cam and Cd-l-Cam crystals, respectively, showcasing the different threshold voltages for spin up and spin down injection. (F) Spin polarization as a function of applied bias voltages for Cd-d-Cam (red) and Cd-l-Cam (blue), respectively. The percentage of spin polarization is calculated as {[(*I*_down_ − *I*_up_)/(*I*_down_ + *I*_up_)] × 100}. Here, *I*_up_ and *I*_down_ are the currents with magnetic north pole up and down, respectively.

For the spin dependent charge transport measurements in chiral 3D MOFs, we have employed the magnetic conducting probe atomic force microscopy (mc-AFM) setup as depicted in Fig. 3A. First, we deposited the crystals onto the gold-coated nickel, Ni/Au (100/8 nm) substrate and the AFM imaging shows the height of the crystals at around 800–900 nm (Fig. 2D) and even more than 1 µm in some cases (Fig. S5). Furthermore, by deploying these crystals, the current *versus* voltage (*I*–*V*) responses under different magnetized conditions *i.e.* magnetic north up and down conditions have been recorded. To control the magnetization direction of the Ni layer, a permanent magnet having a magnetic field strength of ∼0.15 T was placed beneath the ferromagnetic substrate. The measurements were carried out in contact mode using a conductive AFM probe, with an electrical bias ranging from −2 V to +2 V applied to the substrate. Here, when electrons are transported through the multidimensional helical pathway of a 3D MOF, the chirality of the MOF and the coupling of the electron's angular momentum with its spin come into effect. During this propagation of electrons through the chiral pathway, spin–orbit coupling along with the tunnelling assisted charge transport (fundamentally modified by decoherence) generates a spin-dependent energy splitting, thus favouring transport of one spin orientation over the other depending on the chiral handedness.^17^ In the case of Cd-d-Cam, there is a constant observation of higher current when the substrate was magnetized in the UP (green) direction rather than the DOWN (purple) direction (Fig. 3B and S6). Contrarily, for Cd-l-Cam higher current intensity has been observed when the substrate was magnetized in the DOWN (purple) direction rather than the UP (green) direction (Fig. 3C and S6). It has been clearly indicated by the slope of the curves that the current intensity transmitted through the chiral MOF is highly dependent on the spin polarization direction of the injected electrons, which eventually leads to the chirality induced spin selection phenomena. The spin polarization is expressed as SP = {[(*I*_down_ − *I*_up_)/(*I*_down_ + *I*_up_)] × 100}, where *I*_up_ and *I*_down_ represent the currents at a given potential with magnetic north pole up and down, respectively. For Cd-d-Cam, the calculated spin polarization is 81% while for Cd-l-Cam, the calculated spin polarization is 80% at 2 V. From the log plots for Cd-d-Cam, the threshold voltage for down spins exceeds that for up spins (Fig. 3D). Conversely, for Cd-l-Cam, the threshold voltage for up spins exceeds that for down spins (Fig. 3E). It reveals that each spin has a distinct threshold voltage with no evidence of spin flipping during the conduction process therefore holding significance for the CISS effect even at extremely low voltages. It has to be noted that mc-AFM is a completely localized technique where a very fine cantilever tip with the radius in the nanometre scale contacts the sample through the probe–sample contact area and then the respective *I*–*V* curves at different positions are recorded. Additionally, we performed the thickness-dependent CISS studies on the single MOF crystals by deliberately choosing crystals of varying thicknesses. Our results demonstrate that the spin filtering efficiency increases consistently with thickness (Fig. S7). The thickness dependence of spin polarization arises from two competing processes: an increase in spin selectivity due to the growing number of chiral barrier layers and spin relaxation effects caused by spin decoherence effects upon diffusion in the chiral layers. As the thickness of the transporting chiral layer increases, the spin polarization increases because electrons have more opportunities to traverse longer distances through chiral potential, thereby enhancing the spin-filtering process. In our studies, we found that the spin polarization increased with thickness up to 800 nm, beyond which it reached saturation. This can be attributed to an increased likelihood of spin dephasing and scattering at greater thicknesses, which can significantly diminish the observed spin selectivity.^28,39,40^

**Fig. 4:**
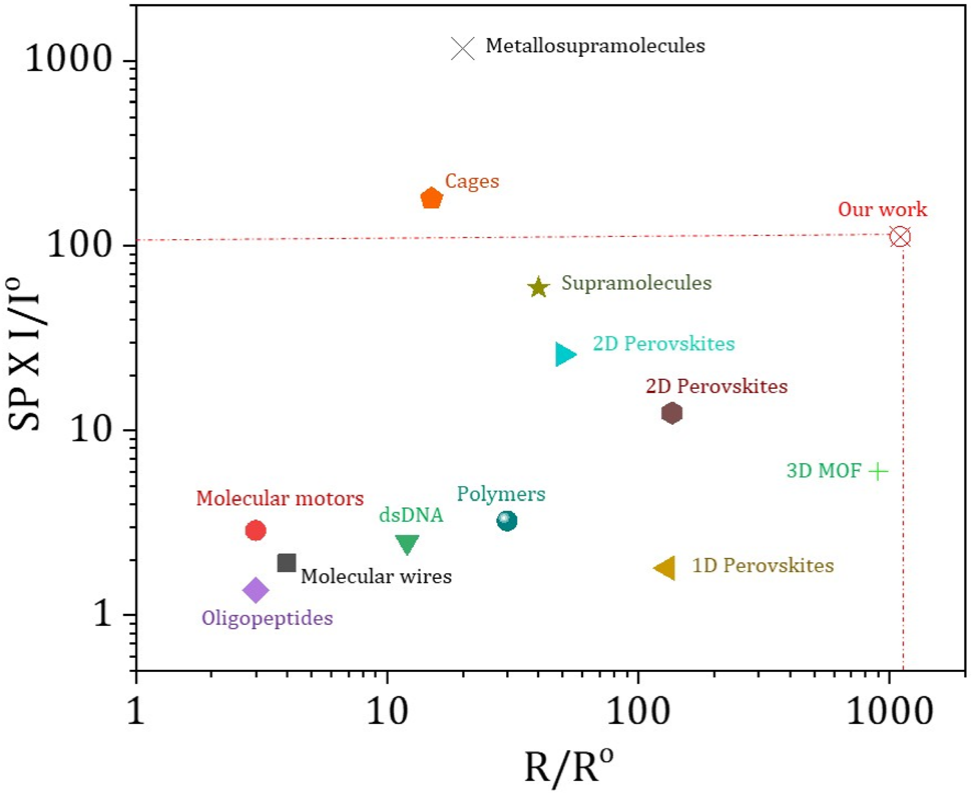
Representation of spin polarization (SP) with *I*/*I*^o^ (*I* is current intensity achieved in nA at the bias voltage of 2 V and *I*^o^ is standardised at 1 nA for all systems) against the *R*/*R*^o^ value (*R* is the range of spin filtering and *R*^o^ is standardised at 1 nm for all systems). Data have been extracted only from representative chiral systems measured by mc-AFM. Data extracted for 3D MOF, see ref. 37; 1D perovskites, see ref. 42; 2D perovskites, see ref. 28 and 43; supramolecules, see ref. 21; metallo-supramolecules, see ref. 19; chiral cages, see ref. 41; polymers, see ref. 20; peptides and DNA, see ref. 40; molecular motors, see ref. 26; molecular wires, see ref. 44. Corresponding FOM values calculated for all these molecular systems are provided in Table S3.

In prior research, chiral systems have primarily been evaluated for their efficiency as spin-filters based on their spin polarization values. As a result, certain systems have been proposed as ideal spin filters, capable of achieving up to 100% spin polarization.^37^ However, important factors such as the current intensity and range of the electron conduction pathway have often been overlooked when evaluating the spin-filtering performance. To ensure a more comprehensive evaluation, we should consider spin-polarization (SP) with the corresponding current intensity (*I*) and conduction range (*R*) as an important figure of merit (FOM) *i.e.* FOM = SP × *I* × *R*. To get a physically significant FOM value, we have redefined the figure of merit where we have converted both the current and spin conduction range into unitless quantities, so that the “figure of merit” will be dimensionless. We express the current intensity as *I*/*I*^o^ and the range of spin filtering as *R*/*R*^o^, with *I*^o^ and *R*^o^ standardized at 1 nA and 1 nm, respectively, across all materials for uniform comparison. The updated figure of merit is now defined as FOM = SP × *I*/*I*^o^ × *R*/*R*^o^. From Fig. 4, it is evident that our current 3D helical MOFs exhibit the highest FOM values, highlighting them as exceptionally efficient spin-filtering materials. Data have been extracted only from representative chiral systems measured using mc-AFM measurements. In Table S3, the data table showcases each parameter of the FOM and the value of the FOM calculated for each molecular system, and we can clearly see the highest value of FOM calculated for our 3D helical MOF system. It is clear that most of the systems attain high spin polarization, while their current intensities are relatively poor. In some cases, particularly in chiral cages^41^ and metallo-supramolecules,^19^ excellent spin polarization is observed with higher current intensity, although the range of the spin conduction pathway is quite shorter than what we have achieved in our work. In our study, we have successfully attained a notable spin polarization (∼81%) and high current intensity (>130 nA) with a long-range spin filtering ability (>1 µm). By combining these three parameters, we can therefore assert that our 3D helical MOF system functions as a highly efficient and long-range spin-filter.

**Fig. 5:**
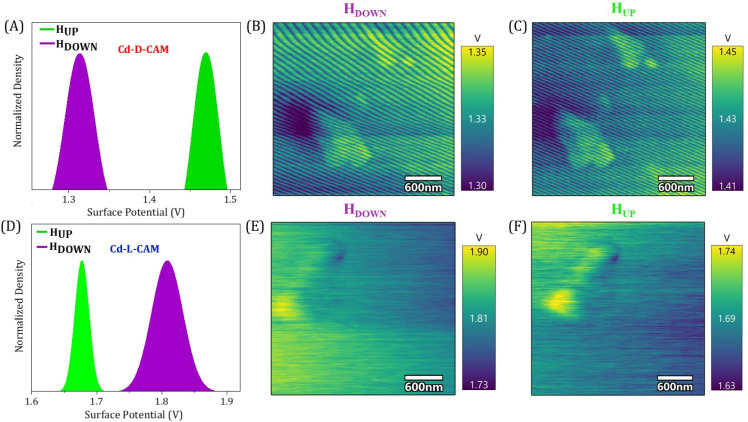
(A and D) Measured contact potential difference (CPD) distribution are shown for Cd-d-Cam and Cd-l-Cam, respectively, with the ferromagnetic layer magnetized with the north pole pointing up (green) or down (purple). (B and C) Electrostatic potential map of Cd-d-Cam with the Ni substrate magnetized with the north pole pointing down and up orientation, respectively. (E and F) Electrostatic potential map of Cd-l-Cam with the Ni substrate magnetized with the north pole pointing down and up orientation, respectively.

We have further employed the Kelvin-probe force microscopy (KPFM) technique to further strengthen the above observations of spin selectivity in chiral 3D MOFs. Here, when charge is injected at the interface from the ferromagnet to the chiral crystals while altering either the chirality (d/l) or magnetization direction (UP/DOWN), spin-selective interactions at the interface of the chiral crystal and the ferromagnetic substrate take place, where charge polarization is accompanied by the spin-polarization thus leading to differential charge accumulation at the surface followed by the change in contact potential difference (CPD).^24,45–47^ In the case of Cd-d-Cam, the CPD is 115 mV higher under magnetic UP conditions rather than the DOWN conditions, as seen in Fig. 5A. Conversely, in the case of Cd-l-Cam, the CPD is 130 mV higher under magnetic DOWN conditions rather than the UP conditions (Fig. 5D). It has also been clearly observed from the potential mapping images (Fig. 5B–F) that the CPD responses are dependent on the chirality of crystals as well as the magnetization direction. The alterations in CPD that arise from the opposite chirality of crystals are related to the penetration of spin-polarized charge from the ferromagnet source into the chiral framework. This effect is facilitated by the CISS effect, which selectively filters electrons according to their spin orientation, leveraging the material's chirality.

**Fig. 6:**
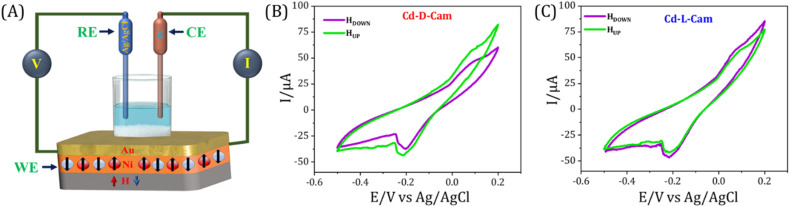
(A) Spin-dependent electrochemical setup for recording current *vs.* voltage (CV) curves under different magnetization conditions. CV curves of the 1 mM K_4_[Fe(CN)_6_]/K_3_[Fe(CN)_6_] redox couple in a pH = 7 (Tris)-buffered, 50 mM NaCl, and 50 mM MgCl_2_ base electrolyte, aqueous solution: initial potential = −0.5 V; final potential = +0.2 V; scan rate = 50 mV s^−1^. Pt acts as a counter electrode, and Ag/AgCl/KCl_sat_ acts as a reference electrode. (B and C) CV curves obtained using Cd-d-Cam and Cd-l-Cam, respectively, physisorbed on the Ni/Au working electrode with the Ni layer magnetized with the north pole pointing UP (green) or DOWN (purple).

In order to further showcase the role of spin in the electron transport process through the chiral MOF materials, we have performed the spin-dependent electrochemical analysis. Similar to the electrochemical setup as used previously,^48,49^ the CV curves were recorded while varying the magnetization direction of a nickel (Ni) substrate as well as changing the chirality of materials. The Ni/Au substrate coated with chiral MOFs acts as the working electrode, a Pt wire was used as the counter electrode, and for the reference electrode, a 3M KCl saturated Ag/AgCl electrode has been used. A 1 mM K_4_[Fe(CN)_6_]/K_3_[Fe(CN)_6_] (Fe^2+^/Fe^3+^) redox couple has been used in the current study due to its simple, well-defined redox reaction and well-reported kinetic, electrochemical and thermodynamic parameters. The study was conducted in an aqueous solution at pH 7, using a Tris buffer, with the supporting electrolyte of 50 mM NaCl and 50 mM MgCl_2_. Before the measurements were performed, a permanent magnet was placed directly underneath the working electrode, and the field strength at the Ni surface was determined to be 0.15 T using a digital gauss meter. Fig. 6 presents the current *versus* voltage (*I*–*V*) plots for chiral 3D MOFs deposited on Ni in different magnetization directions. The CV curves exhibit a redox behaviour, having an oxidation and reduction potential curves at +0.08 V and −0.25 V, respectively. The peaks represent the reversible one-electron transfer of the ferrocyanide/ferricyanide couple *i.e.* [Fe(CN)_6_]^3−^ + e^−^ ⇌ [Fe(CN)_6_]^4−^. Adsorption of a micro-crystalline film over the substrate alters the charge transfer kinetics as suggested by the increased peak to peak difference and decreased peak current along with the asymmetric nature of anodic and cathodic peaks. Such behaviour is generally observed during charge transfer processes through adsorbed molecular films.^50,51^ The CV curves recorded in different magnetic field directions show a clear change in current, which indicates that electron transfer through the chiral 3D MOFs is influenced by spin orientation. On applying a fixed potential under both magnetic conditions, a quantitative comparison of the current values has been done. In the case of Cd-d-Cam a higher current value has been observed under magnetic UP conditions, while a lower current value has been observed under magnetic DOWN conditions (Fig. 6B). Conversely, in the case of Cd-l-Cam a higher magnitude of current has been observed under magnetic DOWN conditions rather than the magnetic UP conditions (Fig. 6C). For the control measurements, we also recorded CV curves for the bare Ni surface under both the magnetic conditions to showcase that the CISS phenomenon arises from the chiral MOF molecules and is independent of the substrate used. It is clearly observed that the obtained CV curves for the bare Ni surface under both the magnetic conditions have no observable change in their current values as both the CV curves almost overlap onto each other (Fig. S8). Additionally, it is important to note that the results obtained from spin-dependent electrochemical analysis for the two chiral 3D MOFs are consistent with mc-AFM and KPFM studies.

**Fig. 7:**
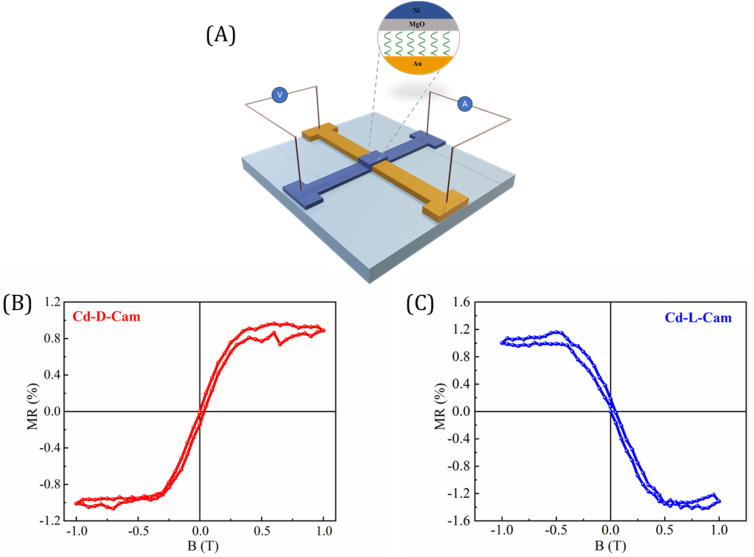
(A) Schematic representation of the four-probe magnetoresistance (MR) measurement setup with the bottom gold and top Ni electrode. (B and C) Magnetoresistance curves as a function of the magnetic field measured with an input current of 0.1 mA at 200 K for Cd-d-Cam and Cd-l-Cam crystals, respectively. MR was measured with an input current of 0.1 mA at 200 K.

Finally, we developed a prototype device in a spin-valve configuration to facilitate the measurement of magnetoresistance (MR) of these chiral MOF crystals. Fig. 7A illustrates the structure of the device, while its crossbar geometry allows for resistance measurements using a standard four-probe configuration. Fig. 7B and C present the MR curves as a function of the magnetic field for devices fabricated from Cd-d-Cam and Cd-l-Cam crystals, respectively. The asymmetric MR curve was observed in response to the magnetic field, which was consistent with previous studies on chiral molecules based on the CISS effect.^27,40^ It is important to emphasize that the results obtained from the MR device not only corroborate the presence of the CISS effect in these studied MOF materials but also align closely with the findings from earlier spin-dependent processes as observed in mc-AFM, KPFM, and electrochemical measurements.

## Conclusion

In conclusion, we have successfully demonstrated chirality induced spin-selective transport in 3D MOFs. Our findings indicate that Cd-d-Cam serves as an effective spin filter for up-spin electrons, while Cd-l-Cam filters down-spin electrons. Notably, we achieved a high spin polarization, along with a substantial current intensity and long-range of spin transport. Based on these three key parameters, we present this work as a pioneering contribution to the field. The helicity manifested in different crystallographic directions offers multichannel electron transport, and the Cd^2+^ metal center may play a significant role in the pronounced spin–orbit coupling in the CISS phenomenon. Furthermore, our report showcases the CISS effect in 3D chiral MOFs using four different techniques, thereby establishing a robust foundation for their applications as long-range spin filters for room-temperature spintronics devices.

## Author contributions

P. S. B. and A. K. M. conceived the idea behind the manuscript. P. S. B., R. G., T. K. D., N. B. and S. C. S. carried out all the experimental works. P. S. B. and A. K. M. carried out the manuscript writing. A. K. M. supervised the project, secured funding, and provided essential resources. All authors contributed to the manuscript through critical review and approved the final version.

## Conflicts of interest

The authors declare no conflict of interest.

## Supplementary Material

SC-OLF-D6SC01358A-s001

## Data Availability

The data supporting the article are provided in the supplementary information (SI). Supplementary information: materials, methods, synthetic details, sample characterization, and additional measurements. See DOI: https://doi.org/10.1039/d6sc01358a.
