# Supplementary material for: Chirality induced long-range spin-selective transport in helical 3D metal–organic frameworks
Source: Chem Sci. 2026 Apr 14;17(21):10735–43. doi: 10.1039/d6sc01358a (PMC13098701; doi:10.1039/d6sc01358a)
Supplement: SC-017-D6SC01358A-s001 [file SC-017-D6SC01358A-s001.pdf]

Supplementary Information (SI) for Chemical Science.
This journal is © The Royal Society of Chemistry 2026

SUPPORTING INFORMATION

Chirality Induced Long-Range Spin-Selective Transport in Helical 3D Metal-Organic Frameworks

Pravesh Singh Bisht,[a] Rabia Garg,[a] Tapan Kumar Das,[b] Nidhi Bhatt,[a] Subash Chandra Sahoo,[c] and Amit Kumar Mondal[a]*

[a] Institute of Nano Science and Technology (INST), Mohali, Sector 81, Sahibzada Ajit Singh Nagar, Punjab 140306, India.

[b] Department of Chemical and Biological Physics, Weizmann Institute of Science, Rehovot 7610001, Israel.

[c] Department of Chemistry, Panjab University Chandigarh, Sector 14, Chandigarh 160014, India.

Corresponding author email id: amit@inst.ac.in

1. Table of contents

1.1 Materials

1.2 Synthesis

1.3 Experimental details

1.1. Materials:

All the solvents and chemicals were purchased from commercial suppliers and used as received. Cadmium nitrate tetrahydrate ($Cd(NO_3)_2 \cdot 4H_2O$) was purchased from LOBA chemie, D/L- camphoric acid (D-H_2cam/ L-H_2cam) was purchased from TCI chemicals, Tetrapyridine-4-yl benzene (TPB) was purchased from BLD chemicals, Dimethyl acetamide (DMA) was purchased from CDH chemicals and all were used without further purification.

1.2 Synthesis:

The MOF crystals Cd-D-Cam were synthesized by the hydrothermal technique using the reported procedure.[1] D-H_2cam (10 mg, 0.10 mmol), TPB (9.5 mg, 0.05 mmol), and $Cd(NO_3)_2 \cdot 4H_2O$ (15.5 mg, 0.10 mmol) were mixed in a Teflon vessel and then 3ml of DMA and 2ml of H_2O was added. The mixture was kept for 3 days at 125°C. Colourless crystals of various shapes and sizes were obtained. These crystals were washed and cleaned with dimethyl

formamide (DMF) and water and further kept in a tightly closed vacuum condition. Similarly, Cd-L-Cam crystals were obtained by using L-H_2cam in place of D-H_2cam.

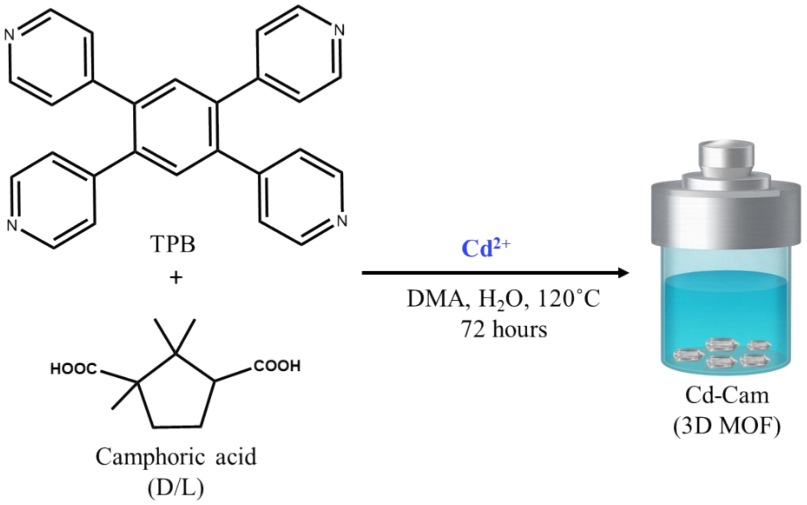

Fig. S1: Synthesis of chiral 3D MOF crystals Cd-D-Cam and Cd-L-Cam.

1.3 Experimental details:

1.3.1 X-ray Crystallography.

Crystals of Cd-D-cam and Cd-L-cam were mounted on Hampton cryoloops. All geometric and intensity data for the crystals were collected using a Super-Nova (Mo) X-ray diffractometer equipped with a micro-focus sealed X-ray tube Mo-Kα (λ = 0.71073 Å) X-ray source, and HyPix3000 detector of with increasing ω (width of 0.3 per frame) at a scan speed of either 5 or 10 s/frame. The CrysAlisPro software was used for data acquisition, and data extraction. Using Olex2,[2] the structure was solved with the SIR2004[3] structure solution program using Direct Methods and refined with the ShelXL[4] refinement package using Least Squares minimization. The crystallographic data and structural parameters were found almost identical with the reported ones[1] (adapted with permission from ref.[1] Copyright 2022, American Chemical Society) and summarized in Tables S1-S2.

Table S1: Crystal Data and Structural Refinement for Cd-L-Cam and Cd-D-Cam.

Crystal Parameter	Cd-L-Cam	Cd-D-Cam
Empirical formula	$C_{69}H_{72}Cd_3N_6O_{13.5}$	$C_{69}H_{75}Cd_3N_6O_{15}$
Formula weight	1538.52	1565.55
Temperature/K	293(2)	293(2)
Crystal system	Trigonal	Trigonal

Space group	$P3_221$	$P3_121$
a/Å	12.6952(2)	12.6854(3)
b/Å	12.6952(2)	12.6854(3)
c/Å	27.5642(5)	27.5641(7)
α/°	90	90
β/°	90	90
γ/°	120	120
Volume/Å^3	3847.29(14)	3841.3(2)
Z	2	2
ρ_{calc}g/cm^3	1.328	1.354
μ/mm^{-1}	0.880	0.884
F(000)	1560.0	1590.0
Crystal size/mm^3	0.01 × 0.006 × 0.004	0.011 ×0.009 × 0.006
Radiation	Mo Kα (λ = 0.71073)	Mo Kα (λ = 0.71073)
2Θ range for data collection/°	6.418 to 54.334	6.424 to 54.282
Index ranges	$-15 \le h \le 15$, $-15 \le k \le 15$, $-34 \le l \le 35$	$-15 \le h \le 15$, $-16 \le k \le 15$, $-34 \le l \le 34$
Reflections collected	42790	22521
Independent reflections	5356 [R_{int} = 0.0648, R_{sigma} = 0.0368]	5234 [R_{int} = 0.0587, R_{sigma} = 0.0605]
Data/restraints/parameters	5356/0/283	5234/0/283
Goodness-of-fit on F^2	1.115	1.062
Final R indexes [I>=2σ (I)]	R_1 = 0.0391, wR_2 = 0.1121	R_1 = 0.0507, wR_2 = 0.1301
Final R indexes [all data]	R_1 = 0.0435, wR_2 = 0.1146	R_1 = 0.0633, wR_2 = 0.1384
Largest diff. peak/hole / e Å^{-3}	1.02/-0.40	0.98/-0.45
Flack parameter	-0.070(14)	-0.07(2)

Table S2: Bond lengths and bond angles in Cd-L-Cam and Cd-D-Cam.

Bond lengths (Å) in Cd-L-Cam and Cd-D-Cam

Cd-L-Cam		Cd-D-Cam	
**Bond Lengths**	**(Å)**	**Bond Lengths**	**(Å)**
Cd01N1	2.299(5)	Cd1N1	2.300(7)
Cd01O2	2.252(5)	CdlO2	2.24 9(6)
Cd01O1	2.473(5)	Cd1O1	2.469(7)
Cd01N2	2.293(6)	Cd1N2	2.286(8)
Cd01O4	2.321(7)	Cd1O4	2.318(9)
Cd01O3	2.383(7)	Cd1O3	2.393(9)

Bond angles (°) in Cd-L-Cam and Cd-D-Cam			
**Cd-L-Cam**		**Cd-D-Cam**	
**Bond Angles**	**(°)**	**Bond Angles**	**(°)**
N1Cd01O1	86.9(2)	N1Cdl01	86.9(3)
N1Cd01C1	102.8(2)	N1CdlC14	102.8(3)
N1Cd01C7	112.0(2)	N1CdlC23	111.8(3)
N1Cd01O4	134.7(3)	N1CdlO4	134.7(3)
N1Cd01O3	88.8(2)	N1CdlO3	88.7(310
O2Cd01N1	117.6(2)	O2CdlN1	117.4(3)
O2Cd01O1	54.65(19)	O2CdlO1	54.6(2)
O2Cd01N2	122.8(2)	O2CdlN22	122.8(3)
O2Cd01C1	27.0(2)	O2CdlC14	26.8(3)
O2Cd01C7	98.2(2)	O2CdlC23	98.5(3)
O2Cd01O4	95.0(3)	O2CdlO4l	95.1(4)
O2Cd01O3	99.7(3)	O2CdlO3	99.7(4)
O1Cd01C1	27.69(19)	O1CdlC14	27.9(2)
O1Cd01C7	152.7(2)	O1CdlC23	152.9(3)
N2Cd01N1	98.78(19)	N2CdlNI	98.8(2)
N2Cd01O1	87.9(2)	N2CdlO1	87.6(3)
N2Cd01C1	107.4(2)	N2CdlC14	107.4(3)
N2Cd01C7	107.5(2)	N2CdlC23	107.6(3)
N2Cd01O4	87.1(2)	N2CdlO4	87.4(3)
N2Cd01O3	125.1(3)	N2CdlO3	125.2(3)

C7Cd01C1	125.2(2)	C14CdlC23	125.2(3)
O4Cd01O1	138.3(3)	O4CdlO1	138.4(4)
O4Cd01C1	118.2(3)	O4CdlC14	118.1(4)
O4Cd01C7	27.3(2)	O4CdlC23	27.2(3)
O4Cd01O3	53.5(3)	O4CdlO3	53.3(3)
O3Cd01O1	147.0(3)	O3CdlO1	147.1(4)
O3Cd01C1	123.7(3)	O3CdlC14	123.7(4)
O3Cd01C7	26.2(2)	O3CdlC23	26.1(3)

1.3.2 UV-Visible Absorption Spectrum.

The Shimadzu UV-2600 spectrophotometer was used to record UV-vis measurements, in the wavelength range of 250–450 nm. For the absorption studies, the solid-state UV-Visible spectroscopy was done using $BaSO_4$ pellets in solid state assembly, where the Cd-L-Cam and Cd-D-Cam crystals were placed on top of $BaSO_4$ and absorption spectrum was recorded.

1.3.3 Circular Dichroism (CD).

Circular Dichroism spectra were recorded using JASCO J-1500 Spectrometer, Easton, MD, USA. Crystal samples were positioned on the quartz plate of 1mm thickness and the samples were measured at 250 nm to 450 nm wavelength range with bandwidth of 2 nm and scan speed of 200 nm min^{-1}.

1.3.4 Scanning Electron Microscopy (SEM).

SEM images were obtained using a JEOL JEM 2100 (Tokyo, Japan) scanning electron microscope at accelerating voltages not exceeding 15 kV, along with Energy Dispersive X-ray Analysis (EDX) pattern for elemental mapping. We placed the crystal on the silicon substrate and allowed the sample to dry under vacuum for 2 hours.

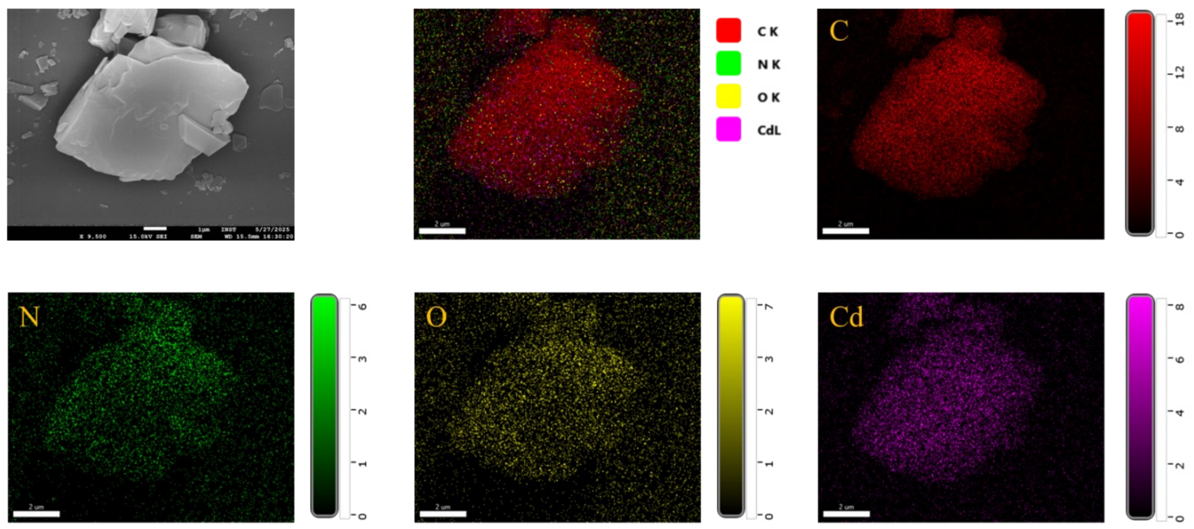

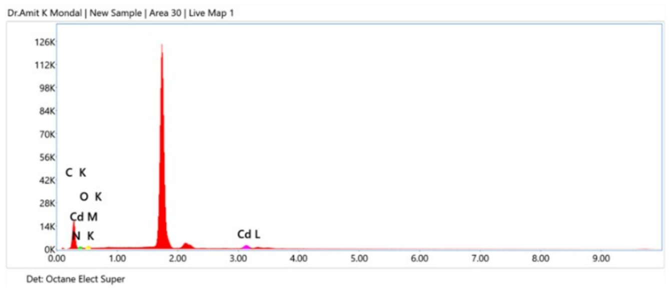

Element	Weight %	Atomic %
C K	68.9	80.1
N K	12.3	12.2
O K	7.1	6.2
Cd L	11.7	1.5

Fig. S2: Elemental mapping of C, N, O, Cd and EDX pattern for chiral 3D MOF.

1.3.5. X-ray Photoelectron Spectroscopy (XPS).

X-Ray Photoelectron Spectroscopy (XPS) using K-Alpha 1063 spectrometer has been used for the elemental analysis of the MOF. The XPS survey spectrum have the peaks of Cd 3d, C 1s, N 1s, O 1s. Here, Cd exists in +2 oxidation state as the high resolution XPS spectrum of Cd 3d has been deconvoluted into two peaks at 412.1 and 405.3 eV which corresponds to Cd $3d^{3/2}$ and Cd $3d^{5/2}$, respectively. C 1s peak has been deconvoluted into 4 peaks i.e. 284.8 eV (C-H/ C-C/ C=C), 285eV (C-N/ C=N), 286.2eV (O-C-O) and 288.1eV (COO^-). N 1s spectrum reveals 2 main peaks positioned at 399.21 and 404.8 eV which corresponds to C=N and N-Cd, respectively. O 1s spectrum reveal 2 peaks at 531.4 eV and 533.22 eV which corresponds to O-C-O and COO-Cd, respectively.[4–7] The elements characterized by XPS align well with the structural formula of MOF.

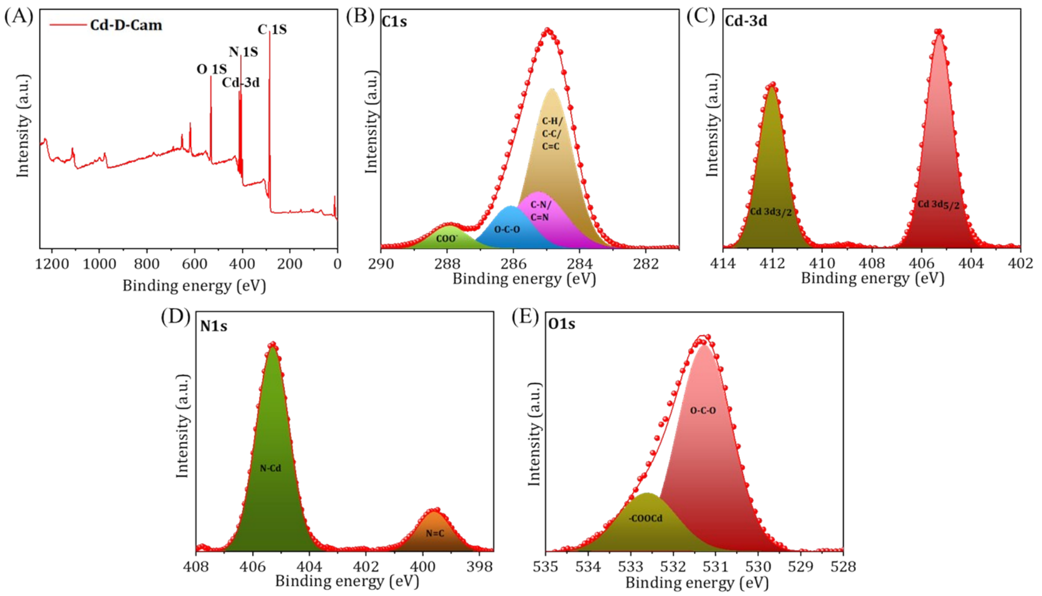

Fig. S3: (A) Survey spectra of Cd-D-Cam 3D MOF crystals. High resolution XPS spectra of (B) C 1s (C) Cd 3d (D) N 1s (E) O 1s.

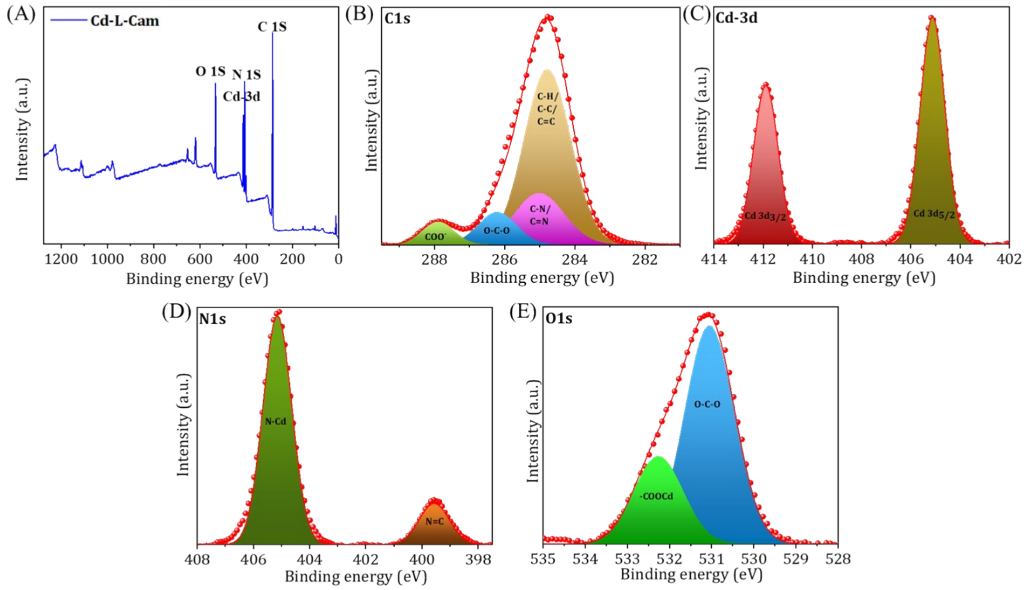

Fig. S4: (A) Survey spectra of Cd-L-Cam 3D MOF crystals. High resolution XPS spectra of (B) C 1s (C) Cd 3d (D) N 1s (E) O 1s.

1.3.6 Atomic Force Microscopy (AFM).

The AFM images were recorded on Asylum Research MFP-3D Origin+Atomic force microscope using Tap300Al-G Tapping Mode AFM Probe with Aluminum Reflective Coating (K~40N/m, F~300kHz) from Budget Sensors and the images were analyzed using AR 19.03.63 analysis software. We took millimetre-sized crystals obtained through solvothermal synthesis

and then further ultrasonicate them in water, which resulted in their breakdown into micro-crystalline forms. The resulting solution was then drop-casted on the substrate and further allowed to dry under vacuum for 2 hours. A film of multiple micro-crystalline crystals has been formed. Height image was obtained using standard tapping mode.

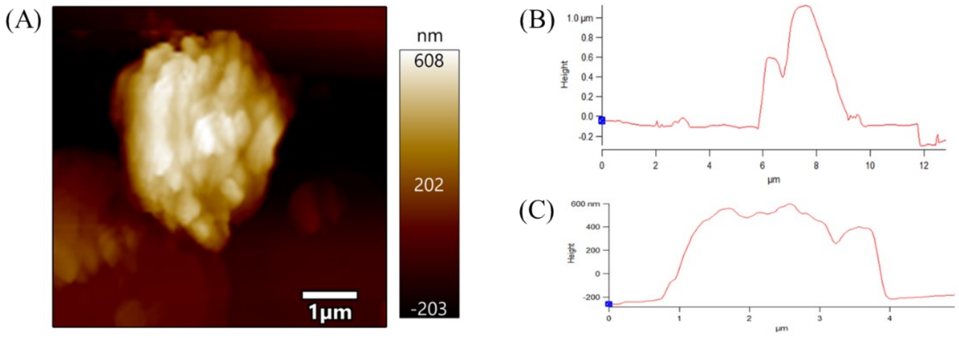

Fig. S5: (A) AFM image of Cd-L-Cam crystal (B) Corresponding height profile image of Cd-L-Cam crystal having height >1μm. (C) Corresponding height profile image of Cd-D-Cam crystal having height ~900 nm.

1.3.7 Magnetic Conductive Probe Atomic Force Microscopy (mc-AFM) sample preparation.

The Ti/Ni/Au surfaces with the thickness of 10/100/8 nm were prepared by sputtering on a Si/SiO_2 wafer for the magnetic conductive probe atomic force microscopy (mc-AFM) measurements. All surfaces were cleaned by immersing them first in boiling acetone and then in ethanol for 10 min. each, followed by a UV-ozone cleaning for 20 min and a final incubation in warm ethanol for 40 min. A film of micro-crystalline crystals was formed over the substrate using same strategy as used for AFM analysis.

1.3.8 Magnetic Conductive Probe Atomic Force Microscopy (mc-AFM).

Magnetic field dependent conductive AFM measurements (I-V measurements) were performed using the MFP-3D Origin Plus under ambient conditions. The bottom electrode i.e. Ni layer was magnetized by permanent magnet of ~0.15 T magnetic field (measured using a Gauss meter), placed under the substrates. The I–V measurements were recorded in the contact mode with a conductive probe ElectriMulti75-G (spring constant is 3 N m^{-1}), with sweeping the biased voltage in the range of ±2 V. The applied force between the tip and the samples was kept at 10-15 nN not to give any damage to the molecules. In order to avoid damaging the tip due to the crystal's height, the scan rate was maintained at 0.5 Hz. The I–V measurements were recorded in different areas of the crystals under up and down magnetized conditions. For each

I–V measurement, the tip was placed in a new position, and movement between points was done with the tip lifted from the surface to prevent damage to the sample and the tip.

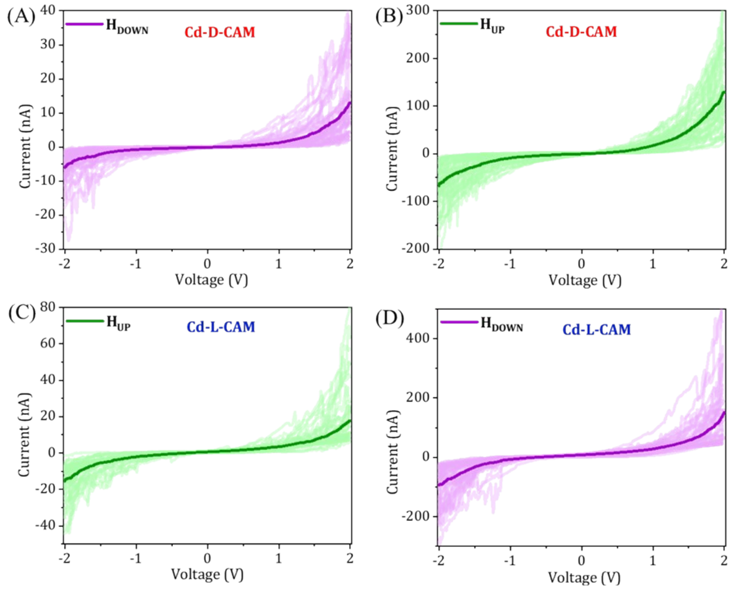

Fig. S6: Spin-dependent conduction study by mc-AFM. (A, B) Current versus voltage (I-V) plots were recorded for Cd-D-Cam. (C, D) Current versus voltage (I-V) plots were recorded for Cd-L-Cam, where Ni substrate magnetized with the North Pole pointing DOWN (purple) and UP (green) orientations.

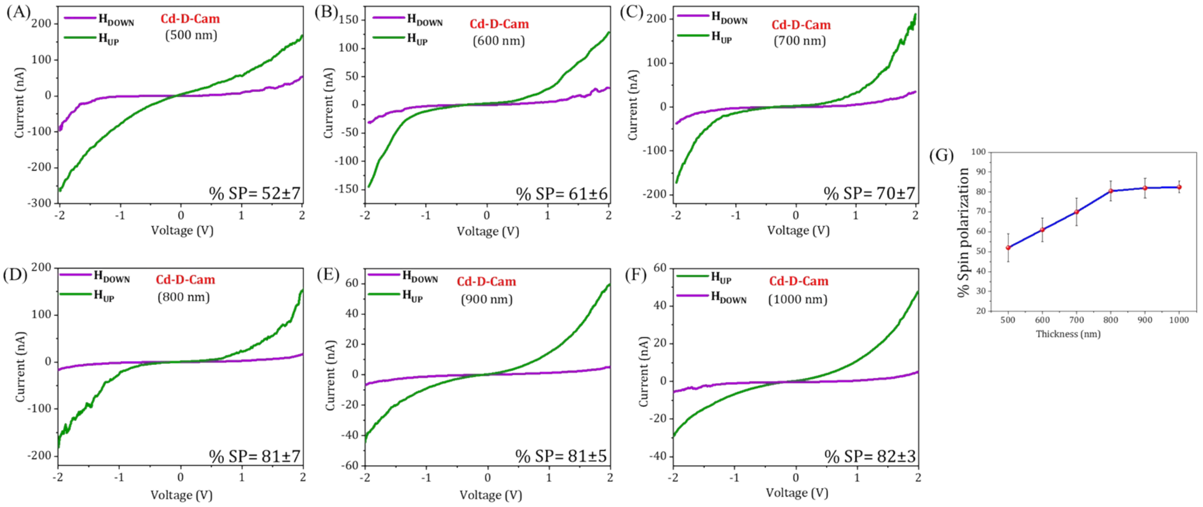

Fig. S7: Spin-dependent conduction study by mc-AFM in Cd-D-Cam crystals having different thickness of single crystals. The averaged current versus voltage (I-V) curves recorded for Cd-D-Cam crystals having (A) 500 nm thickness, (B) 600 nm thickness, (C) 700 nm thickness, (D) 800 nm thickness, (E) 900 nm thickness, (F) 1000 nm thickness with the Ni layer magnetized with the North Pole pointing up (green) or down (purple). (G) A curve showing the dependence of % spin polarization with the thickness of the single crystals.

Table S3: Data table showcasing each parameter of the FOM and the value of FOM calculated for each molecular system.

Molecular systems	Spin polarization (SP)* at 2V	Current intensity (I/I^o) (I^o is standardised at 1nA)	SPXI/I o	Range of spin filtering (R/R^o) (R^o is standardised at 1nm)	FOM= SP × I/I^o × R/R^o
3D MOF	1	6	6	900	5.4×10^3
Twisted molecular wires	0.32	6	1.92	4	7.68
Molecular motors	0.96	3	2.88	3	8.64
dsDNA	0.624	4	2.496	12	29.95
Oligopeptides	0.454	3	1.362	3	4.08
1D Perovskite	0.9	2	1.8	130	234
2D Perovskite	0.86	30	25.8	50	1.29×10^3
2D perovskite	0.83	15	12.45	136	1.69×10^3
Self-assembled Supramolecules	0.59	100	59	40	2.36×10^3
Chiral cages	0.9	200	180	15	2.7×10^3
Supramolecular Polymers	0.65	5	3.25	30	97.5
Metallo-supramolecules	0.9	1300	1170	20	23.4×10^3
Current Work(3D MOF)	0.8	137	109.6	1100	12×10^4

*Spin polarization (SP) = $[(I_{down} - I_{up})/(I_{down} + I_{up})]$

1.3.9 Kelvin Probe Force Microscopy (KPFM).

KPFM measurements were performed using an Asylum Research MFP-3D Origin+Atomic force microscope. The experiments were conducted under ambient conditions. Electric cantilevers, ElectriMulti75-G Force Modulation AFM Probe with Platinum Overall Coating (K~3 N/m, F~75 kHz) from Budget Sensors, were used to conduct the experiments. The surface potential measurements were performed at a 40 nm distance from the surface and the sample was not grounded. For the magnetic field experiments, a permanent magnet was placed underneath the substrate such that 0.15 T magnetic field (measured using a Gauss meter) was applied to the substrate. A film of micro-crystalline crystals was formed over the Ni/Au coated substrate using same strategy as used for AFM analysis. The collected data are used for measurements using repeatable KPFM scans because the measurements are extremely sensitive to dust and ambient factors.

1.3.10 Substrate Preparation for Cyclic voltammetry.

Ni thin film of about 100-120 nm and Au film of about 10-15 nm was deposited on Si wafers by an e-beam evaporator at a pressure of 10^{-5} Torr in a clean room at room temperature. The power of the beam and deposition rate has been adjusted accordingly in order to form a uniform layer. Chiral 3D MOF crystals were dispersed in DI water and sonicated for around 1 hour to break the larger crystals into smaller ones. After that 150μL of the suspension mixed with the 10μL nafion, has been drop-casted on the Ni/Au coated Si substrate and left for drying under IR lamp for an hour. A layer of micro-crystalline sample has been formed and left for overnight drying under vacuum.

1.3.11 Cyclic Voltammetry (CV).

Electrochemical measurements were conducted using a three-electrode system with an Autolab multichannel M 204 PGSTAT (Metrohm) electrochemical workstation. All electrochemical performances were evaluated at room temperature. The Ni/Au substrate coated with chiral MOFs act as working electrode, Pt wire used as a counter electrode and for the reference electrode, a 3M KCl saturated Ag/AgCl electrode has been used. A 1mM $K_4[Fe(CN)_6]/K_3[Fe(CN)_6]$ (Fe^{2+}/Fe^{3+}) redox couple has been used in current study. The study was conducted in an aqueous solution at pH 7, using a tris buffer, with the supporting electrolyte of 50 mM NaCl and 50 mM $MgCl_2$. Before the measurements were made, a permanent magnet was placed directly underneath the working electrode and the field strength at the Ni surface was determined to be 0.15 T using a digital Gauss meter. The Ni surface was secured to the bottom of a teflon cell through an O-ring of 1 cm diameter. The glassware and teflon cell used in the electrochemical experiments, were cleaned by carefully washing in "piranha solution."

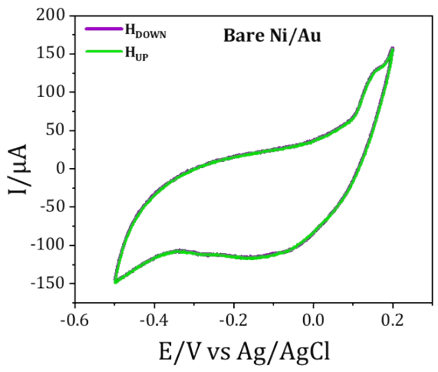

Fig. S8: CV curves under different magnetized condition obtained using bare Ni/Au surface as a working electrode.

1.3.12 Substrate Preparation for magnetoresistance device.

The device used for magnetoresistance studies was fabricated following the procedure described below. The device features a vertical design. The first layer is Au with Ti as an adhesive layer on top of the Si/SiO_2 substrate (111). Optical lithography was used to create Au and Ti lines of 4 μm in width and 80 nm and 8 nm in thickness, respectively. A solution of micro-crystalline MOF crystals was drop-casted onto the gold line and further dried in vacuum. Layer of 2.5 nm thick MgO was deposited by thermal evaporation. The top metal film was composed of Ni covered with Au. The layers were evaporated using a shadow mask with a line width of 50 μm and thicknesses of 80 nm of Ni and 20 nm of Au, in a cross-geometry structure relative to the bottom Au layer. The device was then bonded to a chip carrier and electrically linked using a bonder. The sample was analyzed with a 2T cryogenics system (Cryogenics Ltd.). A magnetic field of up to ±1 T was applied perpendicular to the sample plane. The device's resistance was tested using a typical four-probe approach. DC current of 0.1 mA was applied using a Keithley current source (Model 2400) and the voltage across the junction was measured using a Keithley nanovoltmeter (Model 2182A).

References:

1 P. F. Gao, Y. Y. Jiang, H. Liu, M. S. Zhou, T. Li, H. R. Fu, L. F. Ma and D. S. Li, *ACS Appl. Mater. Interfaces* 2022, **14**, 16435-16444.

2 O. V. Dolomanov, L. J. Bourhis, R. J. Gildea, J. A. K. Howard and H. Puschmann, *J. Appl. Cryst.*, 2009, **42**, 339–341.

3 M. C. Burla, R. Caliandro, M. Camalli, B. Carrozzini, G. L. Cascarano, L. De Caro, C. Giacovazzo, G. Polidori, D. Siliqi and R. Spagna, *J. Appl. Cryst.*, 2007, **40**, 609–613.

4 G. M. Sheldrick, *Acta Cryst. C*, 2015, **71**, 3–8.

5 S. Bhunia, D. Sahoo, S. Maity, B. Dutta, S. Bera, N. B. Manik and C. Sinha, *Inorg. Chem.*, 2023, **62**, 11976–11989.

6 M. Zhang, Q. Yin, X. Ji, F. Wang, X. Gao and M. Zhao, *Sci. Rep.*, 2020, **10**, 3285.

7 S. Ma, L. Gao, Y. Zhang, J. Zhang and T. Hu, *J. Solid State Chem.*, 2022, **306**, 122718.

8 N. Wu and Z. Li, *Chem. Eng. J.*, 2013, **215–216**, 894–902.
